# Secretory IgA in Intestinal Mucosal Secretions as an Adaptive Barrier against Microbial Cells

**DOI:** 10.3390/ijms21239254

**Published:** 2020-12-04

**Authors:** Bernadeta Pietrzak, Katarzyna Tomela, Agnieszka Olejnik-Schmidt, Andrzej Mackiewicz, Marcin Schmidt

**Affiliations:** 1 Department of Food Biotechnology and Microbiology, Poznan University of Life Sciences, 48 Wojska Polskiego, 60-627 Poznań, Poland; agnieszka.olejnik-schmidt@up.poznan.pl; 2 Department of Cancer Immunology, Chair of Medical Biotechnology, Poznan University of Medical Sciences, 8 Rokietnicka Street, 60-806 Poznań, Poland; ktomela@gmail.com (K.T.); mackiewicz.aa@gmail.com (A.M.); 3 Department of Diagnostics and Cancer Immunology, Greater Poland Cancer Centre, 15 Garbary Street, 61-866 Poznań, Poland

**Keywords:** secretory immunoglobulin A, gut, microbiota, immune homeostasis, mucosal secretions, tolerance

## Abstract

Secretory IgA (SIgA) is the dominant antibody class in mucosal secretions. The majority of plasma cells producing IgA are located within mucosal membranes lining the intestines. SIgA protects against the adhesion of pathogens and their penetration into the intestinal barrier. Moreover, SIgA regulates gut microbiota composition and provides intestinal homeostasis. In this review, we present mechanisms of SIgA generation: T cell-dependent and -independent; in different non-organized and organized lymphoid structures in intestinal lamina propria (i.e., Peyer’s patches and isolated lymphoid follicles). We also summarize recent advances in understanding of SIgA functions in intestinal mucosal secretions with focus on its role in regulating gut microbiota composition and generation of tolerogenic responses toward its members.

## 1. Introduction

Immunoglobulin (Ig) is a protein composed of two identical heavy (H) and light (L) chains connected via disulfide bonds. Both chains are composed of variable (V) domains and constant (C) domains. Functionally, Ig is divided into the antigen-binding fragment (Fab) region (paired V_HL_-domains responsible for specific epitope binding with C_L_ and C_H_1 domains) connected through a hinge region to the crystallizable region fragment (Fc, made with remaining C_H_ domains). Differences between Fc constant domains enable an immunoglobulin classification to five isotypes: IgG, IgA, IgM, IgE and IgD [1].

Immunoglobulin A (IgA) is present in all mammals and birds. It is found in large amounts in the mucosal secretions of gastrointestinal tract and in other secretions, including saliva and breast milk [1,2]. However, IgA is also present in serum at lower concentration (about 2–3 mg per mL) [2,3]. In humans, daily IgA production is higher than any other immunoglobulin isotype (up to ~60 mg per kg of body weight) [4].

Monomeric IgA is present in serum, whereas in mucosal secretions is found secretory IgA (SIgA). It is different from the structure of IgA present in the serum because SIgA generally occurs in a polymeric form stabilized by joining chain (J chain), in particular in dimeric or tetrameric setup. Additionally, SIgA contains a secretory component (SC) derived from polymeric Ig receptor (pIgR) utilized for transcytosis through epithelial cells during secretion [2,5]. In humans, there are two subclasses of IgA: IgA1 and IgA2 [6]. In serum subclass IgA1 dominates, whereas in mucosal secretions the proportion between IgA1 and IgA2 depends on the site of production, e.g., up to: 60% IgA1 in saliva, 90% IgA1 in nasal and 60% IgA2 in intestinal secretions [5]. In the human colostrum approximately 48% of immunoglobulins correspond to IgA2 and 40% to the IgA1 subclass [7] that confers an adaptation to protect against potentially harmful pathogens, and which is also a way to regulate the colonization of the microbiota in newborns.

Mucosal membranes lining gastrointestinal, respiratory and genitourinary tracts are exposed to permanent contact with a vast variety of microorganisms. The gastrointestinal tract (GIT) is colonized by numerous and diverse microbial communities, up to ~10^14^ microbial cells per gram of colonic content represented by ~500–1000 bacterial species, archaea and fungi [8]. To prevent the invasion of pathogenic microbes and to regulate interactions between host and bacteria, the mucosal immune system is stimulated to produce SIgA [8,9].

In this review, we present mechanisms of SIgA synthesis in intestines and its functions in intestinal mucosal secretions, mainly based on the knowledge obtained from studies on murine models. Mice are the experimental tool of choice for the majority of biomedical research areas and in numerous respects, they mirror human biology remarkably well. As the differences exist, it is important to comprehend them [10]. Recent findings revealed that inbred mice housed under standardized environmental conditions possess an immature immune system similar to newborn babies, whereas feral and pet-store mice with diverse microbial experience are closer to adult humans [11,12,13]. The conversion of data obtained in a murine model to human biology requires close attention to many differences, including those of ontogeny, tissue of origin, and the potential restrictions of both in vitro and in vivo analyses [14].

## 2. Synthesis of SIgA in the Intestines

The synthesis of serum IgA generally occurs in the bone marrow, where the immunoglobulin is produced as a monomer. There are also other places of monomeric IgA production by plasma cells (PCs), e.g., spleen, lymph nodes, peripheral blood or even intestinal lamina propria (LP), however in much lower proportion compared to the production of polymeric IgA [15,16].

The SIgA is secreted mainly by PCs located within mucosal membranes lining the GIT, and in much lower extent, respiratory and genitourinary tracts [2]. Newborn humans and germ-free (GF) mice have few or no IgA-producing plasma cells (IgA^+^ PCs) in the LP. Bacterial colonization induces IgA^+^ PCs generation, and produced IgA includes specificity for the luminal microbiota [6]. The vast majority of IgA^+^ PCs are present (~80%) in LP of intestinal villi [4]. Studies showed that the small intestinal LP contains up to 15-fold more IgA^+^ PCs than the colonic LP [17]. Abundant production of SIgA in the intestines is one of the mechanisms that gut-associated lymphoid tissue (GALT) runs in response to constant contact of gut mucosal membranes with a large number of diverse microorganisms, mainly bacteria and other food-derived antigens [8]. GALT contains various lymphoid structures (germinal centers, GCs) localized within mucosal surfaces of the intestines, such as highly structured Peyer’s patches (PPs) or isolated lymphoid follicles (ILFs), which are places of SIgA origin [18,19,20]. Of note, these lymphoid structures are predominantly associated with the small intestine [17]. Colonic GALT contains less GCs than small intestinal GALT. Interestingly, the increase in the GCs presence in colonic GALT is associated with colon tumors [21].

The IgA^+^ PCs can be created through T cell-dependent (TD, adaptive response) and -independent (TI, innate response) mechanisms in mice and humans [22]. However, a variety of immune and non-immune cells are indispensable in the process of IgA^+^ PCs generation. In humans, B2 cells, which are bone marrow-derived population of B cells, undergo differentiation to IgA^+^ PCs. Of note, in mice, IgA^+^ PCs originate from two B cell populations, i.e., primitive B1 and B2 cells, whereas in humans, there has not been found any direct equivalent to B1 cells so far [6,23].

### 2.1. T Cell-Dependent Synthesis of SIgA

The main sites for the origination of IgA^+^ PCs are GCs of PPs [4]. PPs are huge structures composed of stromal cell (StC) scaffold, a few (>5) B cell follicles, which are separated by areas of T cells and dendritic cells (DCs). Although PPs develop during embryogenesis, production of IgA occurs in response to bacterial colonization of the intestine [8,24]. A significant decrease in the number and size of PP GCs is observed after microbiota depletion caused by antibiotic therapy [25,26]. T cells are involved in the process of IgA production in PP GCs, but also other lymphoid cells (i.e., B cells, microfold epithelial cells (M cells), DCs) and molecules that they express (i.e., activation-induced cytidine-deaminase (AID), transforming growth factor beta 1 (TGF-β1)) are essential [19,22].

**Figure 1:**
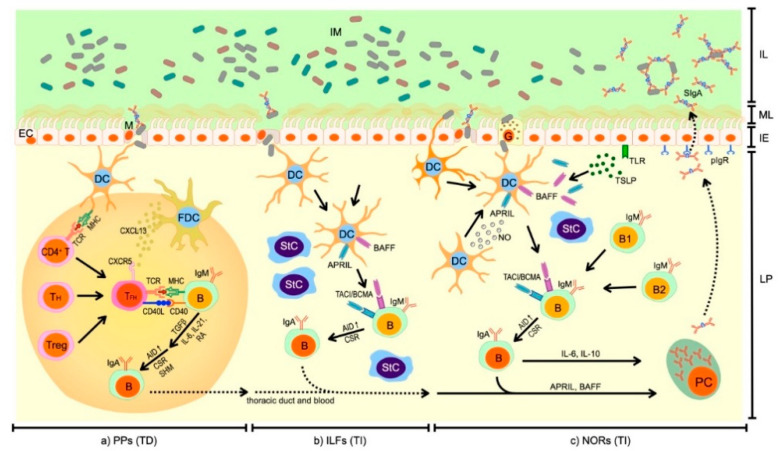
Schematic illustration of T cell-dependent (TD) and -independent (TI) regulation of secretory class A immunoglobulin (SIgA) production in intestinal epithelium (IE) underlying organized structures: (**a**) Peyer’s patches (PPs) and (**b**) isolated lymphoid follicles (ILFs) and (**c**) nonorganized regions (NORs) located in intestinal lamina propria (LP). Signaling pathways between immune and non-immune cells: microfold epithelial cell (M), goblet cell (G), epithelial cell/enterocyte (EC), dendritic cell (DC), follicular dendritic cell (FDC), stromal cell (StC), several types of T cells including: naïve CD4^+^ T cell (CD4^+^ T), follicular helper T cell (T_FH_), regulatory T cell (Treg), and B cells (B) including: primitive B1 and conventional B2 cells, plasma cell (PC), involving signaling molecules: class II major histocompatibility complex (MHC), T cell receptor (TCR), cluster of differentiation 40 (CD40), CD40 ligand (CD40L), interleukin (IL)-21, IL-6, IL-10, transforming growth factor beta 1 (TGFβ), activation-induced cytidine-deaminase (AID), Toll-like receptor (TLR), thymic stromal lymphopoietin (TSLP), a proliferation-inducing ligand (APRIL), B-cell-activating factor of the TNF family (BAFF), C-X-C motif chemokine ligand 13 (CXCL13), C-X-C motif chemokine receptor 5 (CXCR5), nitric oxide (NO) in response to bacterial antigens derived from intestinal microbiota (IM) lead to class-switch recombination (CSR) and somatic hypermutation (SHM) within immunoglobulin loci of B cells followed by production of specific membrane bound immunoglobulins (IgM, IgA) and secretory immunoglobulins (SIgA) within lamina propria and transcytosed via polymeric immunoglobulin receptor (pIgR) by enterocytes to intestinal lumen (IL); mucus layer (ML). Detailed description in text.

The IgA^+^ PCs generation in PP GCs is a complex process (Figure 1a). The response to commensal and pathogenic microbiota begins with sampling of bacterial antigens. M cells localized in follicle associated epithelium (FAE) that is overlying the PP GCs, bind antigens and transcytose them to DCs [27]. DCs migrate to T cell areas where they present antigens and stimulate naïve CD4^+^ T cells to express C-X-C motif chemokine receptor 5 (CXCR5). Subsequently, activated T cells, known as follicular helper T cells (T_FH_ cells) migrate toward C-X-C motif chemokine ligand 13 (CXCL13) to follicular dendritic cell (FDC) network, where they directly stimulate B cells [19,22]. Studies on mice and humans revealed that there are alternative origins of T_FH_ cells. Some of the T_FH_ cells are also generated from helper T cell (T_H_) subsets, such as T_H_1, T_H_2, T_H_17 or regulatory T cells (Tregs) [28]. The T_FH_ cells interact with IgM^+^ naïve B cells through complexes: class II major histocompatibility complex (MHC)–T cell receptor (TCR) and cluster of differentiation 40 (CD40)–its ligand (CD40L). This interaction is crucial for the B cell activation and differentiation into IgA^+^ B cells [19,22]. The CD40L is localized on the T_FH_ cell surface and promotes AID expression in B cells and cooperates with other signals to switch to IgA production [27]. Activated B cells express AID, which is an essential RNA-editing enzyme for class-switch recombination (CSR) and somatic hypermutation (SHM) [4,19,22]. CSR and SHM are two processes, which shape the antibody repertoire at mucosal surfaces [24]. CSR causes alterations in immunoglobulin Fc constant-region gene, resulting in the switch to IgA isotype. Whereas, SHM is a process of accumulation of point mutations in the variable regions of immunoglobulin chain gene, which allow selection of IgA^+^ B cells with higher affinity to antigens [4,24]. TGF-β1 that is produced by B and T cells as well as StCs, FDCs, DCs and epithelial cells (ECs), is a key cytokine regulating the process of IgA switching [22,24,27]. TGF- β1 is produced in latent form and its activation is mediated by matrix metalloproteinases (MMPs), such as MMP9 and MMP13. They are produced by StCs and DCs upon stimulation with tumor necrosis factor alpha (TNFα), produced by DCs and lymphoid tissue-inducer cells (LTi cells) [8]. Additionally, the T_FH_ cells produce interleukin (IL)-21 that has a synergistic activity to TGF-β1 and helps in the proliferation and differentiation of IgA^+^ B cells [22]. Moreover, DCs produce retinoic acid (RA) and IL-6 to induce IgA synthesis [23].

### 2.2. T Cell-Independent Synthesis of SIgA

In mice and humans, precursors of IgA^+^ PCs are also generated in TI manner [22]. For instance, in studies on CD40^−/−^ mice there was not observed any notable decrease in the level of IgA in the gut [29]. Moreover, comparable results were obtained for patients with CD40L deficiency [30]. These data suggest that T cells are not indispensable for IgA production. The ILFs present in murine and human intestinal LP are identified as lymphoid structures that are sites of TI origination of IgA^+^ B cells [19,22]. ILFs, in contrast to PPs consist of StCs and only single separated clusters of B cells, surrounded by a large number of DCs and several T cells [8]. They develop after birth from cell aggregates containing inducer cells (CD4^+^, CD3^−^, CD45^+^, MHCII^+++^), which can be considered the human equivalent of murine cryptopatches [6]. ILF development is stimulated in response to microbial colonization of the intestine as well as to dietary compounds [24,31]. The number, size and composition of ILFs depend on bacterial antigens present in the gut [4].

Despite the fact that the process of IgA^+^ B cells generation in ILFs is better understood in mice than in humans, some aspects are still unclear. Moreover, there are found several differences between murine models and humans. Similar to IgA generation in the PPs, the process starts from the transporting of luminal bacteria to ILFs through M cells present in the intestinal epithelium (Figure 1b). Upon luminal antigens sampling, DCs produce B-cell-activating factor of the TNF family (BAFF), a proliferation-inducing ligand (APRIL) and TGF-β1 that bind to receptors expressed on naïve B cells (respectively to: B cell maturation antigen (BCMA), transmembrane activator and calcium modulator and cyclophilin ligand interactor (TACI), and activin receptor-like kinase-5/transmembrane receptor serine/threonine kinase type II receptor (ALK5/TβRII) [32]) [23]. In mice, but not in humans, B1 cells originating from the peritoneal cavity are predominantly involved in the TI IgA production. They migrate to intestinal LP upon stimulation with Toll-like receptor (TLR) ligands. In humans, B2 cell lineage is solely present and their differentiation into IgA^+^ B cells occurs upon ligation of TACI with APRIL (in humans predominantly produced by ECs) [23]. Studies have demonstrated a decrease in serum IgA level in APRIL- or TACI-deficient mice and in humans with TACI mutations, suggesting their indispensable role in TI IgA generation [33,34,35].

Moreover, studies have shown that IgA^+^ B cells might be also generated in non-organized intestinal LP [19]. As well as in the PPs and ILFs, SIgA synthesis in non-organized intestinal LP starts from bacterial and food-derived antigen sampling. This process occurs through M cells or directly via extended dendrites of DCs through tight junctions and through goblet cells (Figure 1c). Naïve B cell switching to IgA^+^ B cells in the absence of T cells occurs upon stimulation with BAFF and APRIL produced by DCs, StCs and ECs upon stimulation with nitric oxide (NO) and thymic stromal lymphopoietin (TSLP) [8,19,22,23]. Moreover, other factors are also identified as stimulators of TI IgA generation in humans, such as IL-10 and IL-4, retinoic acid (RA), and IgA-inducing protein (IGIP) [21].

However, the role of human non-organized intestinal LP as an inductive site for IgA responses is still unclear. For instance, studies on murine models showed that there is no expression of AID in non-organized LP, which is indispensable for switching to IgA^+^ B cells [29]. Results obtained in previous studies might be a consequence of LP sample contamination with ILFs and this issue is still debated [6].

### 2.3. Migration of IgA^+^ B Cells and Differentiation into IgA^+^ PCs in LP

IgA^+^ B cells migrate from PPs and ILFs to mesenteric lymph nodes (MLNs) via lymphatics. MLNs are the location of IgA^+^ B cell proliferation and differentiation into plasmablasts. Subsequently, plasmablasts home to the gut LP through the thoracic duct and blood [8]. Migration of IgA^+^ PC precursors to LP is stimulated by the interaction between α_4_β_7_ integrin expressed by IgA^+^ B cells and plasmablasts and mucosal vascular addressin cell adhesion molecule 1 (MAdCAM1) produced by endothelial cells in intestinal LP. However, other factors such as thymus-expressed chemokine (TECK) and StCs present in LP are also involved in this process [4]. The expression of gut-homing receptors on lymphocytes is induced by RA [23]. In the LP, plasmablasts differentiate into IgA^+^ PCs upon stimulation by IL-6, IL-10, BAFF and APRIL [8].

In the gut LP, monomers of IgA, produced by PCs, interact with the J chain to form a dimeric structure that can recognize pIgR. Linkage with pIgR enables transport through the ECs to mucosal surfaces. SIgA in the gut lumen contains SC–pIgR-derived polypeptide that stabilizes SIgA in the mucus [36].

## 3. Functions of SIgA in Gut Mucosal Secretions

SIgA is produced in response to microbial- and food-derived antigens and plays different roles in intestinal mucosal secretions (Table 1). It acts as the first line of defense against pathogens and facilitates mucus surface colonization by commensal microbiota and regulates immune homeostasis [24].

Two mechanisms of SIgA binding to antigens are possible: canonical and non-canonical. Canonical (Fab-dependent) SIgA binding to its antigens occurs via the complementarity-determining regions (CDRs), whereas non-canonical via glycan moieties of this molecule. The process of IgA^+^ B cell SHM causes alterations in CDR sequence and structure and leads to the generation of affinity-matured IgA [46]. SHM occurs mainly during T cell-dependent, but also during T cell-independent IgA^+^ B cell maturation [46,47]. Secreted forms of IgA, but also J chain, and SC are heavily glycosylated. O-glycans located between Cα1 and Cα2 domains of SIgA heavy chain are involved in bacteria binding. The sugar moieties also protect the hinge from bacterial protease digestion. The wide range of N-linked glycans on SC target bacterial adhesins as well, and anchor SIgA to the mucosal lining of the epithelium entrapping microorganisms [38].

Pathogenic strains and toxins are predominantly neutralized by high-affinity SIgA produced through TD mechanism, whereas gut commensal microbiota is coated with low-affinity IgA produced through TI mechanism [18]. However, there are some exceptions to this rule. For instance, Bunker et al. (2015) observed that some commensal bacteria, such as segmented filamentous bacteria (SFB, *Candidatus* Arthromitus) and *Mucispirillum* elicited IgA responses in TD manner [17].

According to studies, SIgA exhibits cross-species reactivity. It means that SIgA has the ability to bind to various but specified species of microorganisms [46]. In studies conducted on intestinal IgA plasmablasts isolated from patients with ascending colon cancer, Benckert et al. (2011) observed that about half of IgA clones do not react with a panel of commensal and pathogenic microbial strains, third of the clones were cross-species reactive, fifth were self-reactive and the small fraction of clones reacted with single target of prepared panel of microbial strains [24,48]. However, this ability of SIgA to bind to multiple microbial species might result from its polyreactivity. Highly glycosylated molecules of SIgA can interact with glycans exposed by intestinal bacteria, such as O-antigens, polysaccharide capsules, and teichoic acids [46]. Although the structure of many bacterial surface glycans is unknown, it was observed that almost identical glycan motifs are present on commensal *Escherichia coli* and pathogenic *Haemophilus influenzae.* This result suggests that cross-species binding of SIgA might rely on recognition of similar surface glycans present on even unrelated species [46,49].

### 3.1. Defense against Pathogens

SIgA plays various roles in the gut mucus secretions. Initially, it was thought that SIgA only protects intestinal milieu against bacterial, viral or parasitic mucosal pathogens [8]. Various studies from the 1990s showed that SIgA is secreted in response to strains that cause diseases, e.g., *Campylobacter jejuni*, *Shigella sonnei*, enterotoxigenic *Escherichia coli* or *Aeromonas* species [50,51,52]. It was also shown that SIgA plays a key role in neutralizing bacterial exotoxins, such as *Clostridium difficile* toxin A and cholera toxin [39,43,44].

Defense against pathogenic microorganisms relies on binding of SIgA to pathogens and toxins to prevent adhesion to intestinal tissues through a non-inflammatory mechanism called immune exclusion [37]. This process enables pathogens entrapment in mucus and their removal by peristaltic activities. However, SIgA also prevents pathogen attachment to gut ECs by blocking of receptor-binding domains in Fab-dependent or Fab-independent manner or by indirect steric alteration of these domains [39]. Moreover, SIgA also has the ability to suppress bacterial virulence. Forbes et al. showed that binding of IgA to *Shigella flexneri* antigen caused inhibition of secretion system, which is involved in getting access to the intestinal epithelium [39,45].

Studies have shown that food-derived antigens elicit defense against pathogen infection. Hara et al. observed that GF mice kept on an antigen-free diet (GF-AF) were more susceptible to *Salmonella typhi* serovar Typhimurium infection. However, in GF mice exposed to dietary antigens production of pathogen-specific SIgA was induced. This protective response might result from the induction of PP GCs and increased number of Foxp3^+^ T cells, which are precursors for the T_FH_ cells, as compared to GF-AF mice [53].

SIgA also establishes protection against viral infections of the GIT. Blutt et al. observed retardation in the clearance of viral infection in IgA lacking mice (IgA^−^/^−^) compared to wild-type mice. Although IgA^−^/^−^mice were exposed to multiple contacts with the virus, they did not develop any protective immunity. Moreover, IgA^−^/^−^ mice were more susceptible to rotavirus infection than wild-type mice [54].

Some pathogenic strains such as *Haemophilus influenzae*, *Streptococcus pneumonia, Streptococcus sanguis, Neisseria meningitidis* and *Neisseria gonorrhoeae* produce IgA-specific proteases [5,55]. Moreover, some pathogens inhibit actions of IgA by the production of IgA-or SC-specific binding proteins. SSL7 toxin produced by *Staphylococcus aureus* binds to IgA and prevents its interaction with FcαRI. It is a surface receptor present on immune cells, such as neutrophils, eosinophils, monocytes, macrophages and DCs that in complex with IgA activate immune responses [5]. By mimicking host antigens, other microbial pathogens evade immune surveillance. *Campylobacter jejuni* is a gut pathogen causing acute diarrhea. Lipopolysaccharide (LPS) and lipooligosaccharide (LOS) of the outer core oligosaccharide structures from *C. jejuni* have sialylated moieties with configurations identical to those of several gangliosides [56]. These properties of the pathogen show that SIgA plays an important role in its neutralization. Of note, IgA2, the dominant IgA subclass in the intestinal secretions, has a shorter hinge than IgA1 and is less susceptible to bacterial proteases. Consequently, IgA2 has functional advantages in such a dense and diverse bacterial population that exists in the intestines [21].

Although SIgA agglutination of pathogens and exclusion from mucosal surfaces is a major defense mechanism, SIgA exhibits the potential (in vitro studies) to trap pathogens that have breached the epithelial barrier and expel them from the LP. Also, SIgA can neutralize viruses intracellularly while being transcytosed to the apical surface. In vitro analyses of nasopharyngeal ECs and hepatocytes indicated a possible mechanism that some bacterial, viral and fungal pathogens can hijack the defensive role of SIgA to enhance their own infection by binding to pIgR and SC [57]. However, it remains to be further analyzed especially with respect to gut pathogens.

### 3.2. Regulating Gut Microbiota Composition, Gut Immune Homeostasis and Oral Tolerance

Apart from the pathogen neutralization, SIgA regulates gut microbiota composition and plays the role in maintaining homeostatic relationship between intestinal microbiota and the host [58]. The gut microbiota serves a positive role for its host (reviewed in [59]), therefore keeping the balance of antimicrobial responses has strong evolutionary pressure and involve tolerogenic mechanisms.

Immunological tolerance is a set of mechanisms that prevent potentially harmful immune responses within the host. It comprises among other a removal or inactivation of self-reactive B cells, including those that also recognize cross-reactive foreign antigens. The first (central) tolerance checkpoint occurs in bone marrow, where most of polyreactive B cells and/or those reactive with nuclear antigens are removed. The second (peripheral) tolerance checkpoint removes auto-reactive new emigrant B cells (CD10^+^, CD19^+^, IgM^++^, CD27^−^) that recognize peripheral antigens not expressed in the bone marrow [60]. In case of antigen delivered through the gastrointestinal tract, the mechanism is called oral tolerance. This type of tolerance can lead to the suppression of effector responses throughout the organism [61]. Intestinal responses to orally-delivered antigens are not eliminated, but instead comprise peripherally derived Tregs and the production of SIgA (type 4 response) [62]. As long as the bacteria remain in the gut lumen, a protective tolerogenic response is induced. In situations where pathogenic bacteria invade the epithelium, a proinflammatory signaling cascade is initiated [63]. A key role of the gut immune system is to generate oral tolerance to food antigens and commensal microbiota while preserving the protective activity against pathogens. Therefore, the tolerance is a complex process that involves the coordinated action of both immune and non-immune cells at mucosal sites. The local microenvironment, and especially intestinal ECs, plays a significant role in the induction of tolerogenic state. The ECs release TSLP, RA and TGF-β. These induce a ‘mucosal’ phenotype to DCs (CD103^+^) making them unable to release inflammatory cytokines. TSPL promotes the release of APRIL and BAFF by DCs and generation of protease-resistant IgA2 after sequential class switching from IgA1. Also, macrophage activity is modulated by StC-derived TGF-β and Tregs to drive the monocyte differentiation into anti-inflammatory cells. Tregs execute tolerogenic functions of macrophages through secretion of IL-10 [63,64]. Noble et al. (2020) proposed human gut tissue resident memory T (T_RM_) cells, that express high levels of the Treg markers CD39 and CD73, to be responsible for immunosuppressive function to maintain tissue homeostasis, and also a possible role of CD8^+^ T cells. They provided evidence that human gut T_RM_ preferentially expresses Runx3, and further co-express CD39 and CD73 [65]. CD39 due to its ability to degrade extracellular ATP (a DCs activator [66]) and further activity of CD73 catalyzing nucleotide breakdown to adenosine provide the immunosuppressive molecule [67]. Although Foxp3^+^ Tregs are critical in systemic tolerance [68] their small number in the human gut is insufficient to maintain tolerance to such large microbiota-derived antigenic loads, requiring the acquisition of T_RM_ cells in mucosal tissue [65].

There is a relative lack of studies that investigate regulation of B cell reactivity to commensal bacteria at homeostasis. Bos et al. showed that commensal and pathogenic microbiota induce the SIgA production. They observed that SIgA level in GF mice was scanty, whereas microbial colonization caused its rapid increase [46,69]. Bunker et al. observed that the majority of commensal microbiota that resides in the small intestine induces IgA production, whereas most species indigenous to the colon do not elicit such responses. Moreover, they found that nearly all IgA-coated taxa from colon were also present in the small intestine. These data suggest that IgA responses predominantly target commensal bacteria in the small intestine, whereas colonic IgA-coated taxa represent population originating from the small intestine [17]. However, findings of Chang et al. (2019) provide an example indicating the existence of exceptions to this statement [70].

In studies conducted on AID-deficient mice (in which CSR and SHM mechanisms were disrupted) and AID^G23S^ mice (SHM was disrupted), Wei et al. showed that the absence or even limited repertoire of diversified SIgA caused an enormous expansion of anaerobic microorganisms in the small intestine. Dysregulation of microbial composition in the gut also caused enhanced activation of systemic immune responses [19,71]. Similar results were obtained in studies on programmed cell death-1 receptor deficient mice (PD-1^−^/^−^), in which the selection of IgA precursor cells in the PP GCs was disrupted [19,72]. PD-1 is responsible for the maintenance of T_FH_ cells, and consequently induces a diversified IgA repertoire in the PPs [23]. These results showed that antigen-specific SIgA is indispensable in regulating the composition of gut microbiota and leads to homeostasis by prevention from inflammatory responses [19]. Bacterial pathogen-SIgA immune complexes reduce the expression of pro-inflammatory molecules by DCs. The anti-inflammatory effect is specific to SIgA, as serum IgA-immune complexes enhance production of pro-inflammatory cytokines by monocytes and macrophages [57]. Another anti-inflammatory mechanism of SIgA is mediated by its LPS-specific binding preventing LPS-induced nuclear factor kappa-light-chain-enhancer of activated B cells (NF-κB) translocation [73].

Analysis of such sparse data does not allow for a hypothesis on whether gut Tregs promote and/or inhibit IgA responses [74]. However, the latest research indicated that intestinal Tregs (CD4^+^Foxp3^+^) can promote tolerance toward commensal bacteria, while sustaining immunoreactivity to pathogens. Kuczma et al. (2020) showed in a murine model that *Akkermansia muciniphila* accelerates the conversion of CD4^+^ cells to peripheral Tregs (pTregs) followed by reduction of intestinal inflammation [75]. *A. muciniphila* is a typical member of healthy human gut microbiota, which is often shown to be significantly decreased in various diseases [76], therefore possibly able to protect the host from colitogenic effector cells. Additionally, the microbiota derived metabolites (especially butyrate and propionate) feasibly support the expansion of pTregs [77,78] and promote anti-inflammatory effects [79]. Another example of commensal bacterium that contribute to gastrointestinal health was much earlier provided by Kelly et al. (2004). They found that *Bacteroides thetaiotaomicron* acts on NF-κB, but its mode of action is distinct from that of pathogenic species. Pathogens (e.g., *Yersinia* sp., some *Salmonella* spp. strains) usually inhibit activation NF-κB, whereas *B. thetaiotaomicron* targets transcriptionally active RelA (a NF-κB subunit) in intestinal ECs through a peroxisome proliferator-activated receptor gamma (PPAR-γ)-dependent pathway. This induces premature nuclear clearance and as a result of that shorten the duration of NF-κB action. Thereby, the commensals downregulate proinflammatory cytokine expression conferring anti-inflammatory action [80,81]. The research of Joglekar et al. showed that SIgA directed diverse bacterial antigens against the *B. thetaiotamicron* target, including capsular polysaccharides [82], LPS [83], and proteins. Their most recent research indicated that among microbial protein targeting-SIgA one specificity was identified against proteins of the polysaccharide utilization locus (PUL). The locus encodes activities necessary for utilization of fructan, an important dietary polysaccharide. The expression of PUL is associated with the *B. thetaiotaomicron* colonization of the gut in the presence of dietary fructans. The presence of this dietary polysaccharide increases the production of PUL-specific IgA. The antibody downregulates the expression of PUL in *B. thetaiotaomicron,* thus regulating microbial colonization by modulating its metabolism [42]. This example shows, how SIgA can selectively restrict the microbial metabolic activity and growth, therefore, managing composition of intestinal microbiota and its contribution to mucosal homeostasis.

Sutherland et al. (2016) hypothesize the SIgA produced in TI manner is effective at excluding microorganisms from the intestines, however does not promote mutualism with microbiota in the same way as SIgA produced by PCs originating in TD mechanisms. Their research, reviewed in [84], indicates that adaptive-IgA promotes more advanced mutualism with the microbiota than innate-IgA by selecting and diversifying beneficial microbial communities. Emerging evidence indicates that the most of commensal bacteria-targeting SIgA is produced via innate mechanisms and polyreactive. These TI B cell responses and antibody production are modulated by group 3 innate lymphoid cells (ILC3) via the production of lymphotoxin and B cell survival factors such as BAFF and APRIL [85]. However, some mucosal-dwelling species can also elicit SIgA via TD mechanisms [17]. Recent research of Melo-Gonzalez et al. (2019) suggests that ILC3 acts as an important checkpoint to maintain tissue homeostasis. This is achieved by regulating balanced interactions between T_FH_ and B cells to limit mucosal IgA responses. Antigen presentation by ILC3 to T_FH_ is necessary to suppress the subsequent initiation of IgA^+^ B cell responses against both commensal and pathogenic bacteria residing within the colonic mucosa [40].

Research of Chang et al. (2019) brought interesting results. They analyzed eight different species of human gut commensal bacteria representing the most prominent phyla of the human gut including *Firmicutes*, *Bacteroidetes*, *Actinobacteria* and *Proteobacteria*. Monocolonized GF mice produced SIgA at different levels, however, *Bacteroides ovatus* induced significantly higher SIgA secretion compared with mice colonized with any of the other seven human gut bacteria. Moreover, analysis of 19 *B. ovatus* strains isolated from different individuals and distinct strains of additional common species from the order *Bacteroidales* revealed various strain SIgA-induction capacity with unique high IgA-inducing ability specific to only a subset of *B. ovatus* strains. Even in more complex microbiota compositions *B. ovatus* proved to be a major contributor of gut IgA responses. This indicates that the induction of SIgA secretion is not only species-but also strain-specific. A clear trend towards a positive correlation was noted for robust gut *B. ovatus-*specific IgA-responses also in humans [70]. Surprisingly, inconsistent with other results [17], the SIgA responses against *B. ovatus* were found to be significantly higher in colon than the small intestine. No significant low- vs. high-IgA-inducing *B. ovatus* strain difference was observed in the small intestine, where comparable luminal IgA levels were produced. The gut SIgA production elicited by *B. ovatus* was primarily via TD B cell-activation pathway, however, CD4^+^ T cells were not a dominant factor in the early stage of IgA induction [70]. These data are in agreement with previous findings on the ability of the host to target a precise trait of the bacteria [86,87], not necessarily a taxonomic group, to direct specific selection toward microbiota members.

In steady-state conditions, approximately 36% of the gut microbiota is coated with SIgA, whereas during inflammation, this number can increase up to 69% [88]. SIgA stimulates antigen sampling from the gut lumen and elicits mucosal immune responses to prevent their penetration farther than to the MLNs and systemic dissemination [39]. Complexes of SIgA-coated bacteria are captured and transcytosed by M cells with enhanced efficiency. Primarily, it was thought that dectin-1 expressed by M cells is involved in this process; however, studies showed that sampling of SIgA-antigen complexes occurs also in the absence of this receptor [27]. Moreover, such complexes interact with the intestinal epithelium and cause intestinal barrier enhancement, increased pIgR production and downregulation of inflammatory responses [39]. Interestingly, it was found that intestinal SIgA preferentially coat colitogenic bacteria, therefore preventing the perturbation of enteric homeostasis and inflammation [86,87]. All these mechanisms support commensal microbiota existence in non-inflammatory relationships with the host [19].

SIgA is also crucial in maintaining diverse and stable intestinal microbiota composition in humans. Bollinger et al. observed that SIgA and mucin promote biofilm formation and gut colonization by commensal microbiota [39,41]. Catanzaro et al. showed microbial diversity reduction and alterations in relative abundances of some microbial taxa in patients with selective IgA-deficiency (SIgAd) compared to healthy controls. Moreover, IgM in SIgAd patients was less specific than IgA in healthy controls and bound multiple bacterial targets [89]. In many cases, patients with IgA-deficiency develop gastrointestinal disorders, e.g., celiac disease, allergies [39].

SIgA plays also the key role in regulating intestinal microbiota maturation. Mirpuri et al. showed that *Proteobacteria*-specific IgA stimulates maturation of gut microbiota in mice. In studies conducted on newborn and adult mice, they observed that newborn microbiota, defined as “immature”, were dominated by *Proteobacteria* spp. in particular *Enterobacteriaceae*, whereas adult microbiota, defined as “mature” consisted mainly of *Bacteroidetes* and *Firmicutes.* Additionally, newborn mice had increased levels of proinflammatory cytokines compared to adult individuals. They observed that IgA from colonic fecal content from adult mice exhibited higher reactivity with immature microbiota. Moreover, the colonization of GF mice with newborn microbiota caused production of *Proteobacteria*-specific IgA. However, in IgA^−^/^−^ mice expansion of *Proteobacterium* spp. enhanced proinflammatory responses were observed in newborn and adult. These results suggest that *Proteobacteria* are major inducers of SIgA production. SIgA specific to *Proteobacterium* spp. takes part in regulating maturation of gut microbiota and limits colonic inflammation associated with *Proteobacteria* [90].

These processes provide examples of evolutionary adaptation of host–microbe interactions within the gut ecosystems to maintain homeostasis by attenuating host immune-functions. Both TI and TD B cell regulatory mechanisms are crucial for selecting and maintaining refined bacterial communities with beneficial properties. The most recent findings in the field are summarized in Table 2.

## 4. Conclusions

SIgA plays a crucial role in the mucosal surfaces lining the GIT. A large number of studies have shown that SIgA is involved in maintaining intestinal homeostasis by neutralization of pathogenic microorganisms and toxins, downregulation of inflammatory responses, regulation of gut microbiota composition and protection from improper immune responses to antigens from microbes and food [24]. Despite the fact that there are various studies on mice and humans, some mechanisms, which regulate such SIgA functions, are still unknown. SIgA plays an important role in shaping the gut microbiota structure during newborn colonization that may be crucial for proper systemic immune response development. Research of Baldassarre et al. (2016) showed the possibility of intervention to modulate colonization and microbiota development in neonatal gut by probiotic administration to women during pregnancy and breastfeeding changing the cytokine pattern in milk that enhanced the production of SIgA in neonates [91]. Dysbiosis (e.g., antibiotic induced) at an early ontogenetic stage may be responsible for susceptibility to non-infectious diseases due to shaping aberrant gut-immune axis signaling. Wider studies, which could shed light on these processes are needed. The SIgA plays a key role in host–microbe interaction and the mechanism underlying the selective recognition of gut microbiota will allow understanding of many gastrointestinal and also systemic diseases. The microbiota regulation of B cell activity may also shed a new light on the complex regulatory mechanisms of anti-tumor responses. Several studies have revealed the different roles of two specific B cell subpopulations. The IgG^+^ B cells play an antitumor role via IgG-mediated antigen-presentation by DCs and activation of antitumor T cell responses, whereas the IgA^+^ B cell subpopulation is immunosuppressive because it induces multiple tumor-promotion mechanisms [92,93].

## Figures and Tables

**Table 1:** The primary functions of SIgA in gut mucosal secretions.

Function	Mechanism	Reference
Immune exclusion	Retention of bacteria in the intestinal lumen.	[8,18,37]
Niche occupancy	Binding bacteria to mucus.	[18,38,39,40,41]
Immobilization	Impairing mobility by targeting flagella, cell agglutination.	[18]
Modulation of bacterial gene expression and metabolism	Targeting bacterial surface end extracellular proteins.	[42]
Neutralization	Targeting adhesins and toxins.	[8,18,39,43,44,45]
Antigen uptake	Facilitated transcytosis.	[27,39]

**Table 2:** Summary of recent findings on SIgA in mucosal secretions.

Finding	Reference
Infections and immunizations modulate maternal SIgA at the mammary gland impacting the development and protection of the newborn.	[7]
Dietary antigens are essential for a normal antigen-specific IgA response to bacteria.	[53]
T_RM_ and its interaction with DC contribute substantially to the B cell activity.	[65]
The ability and localization of SIgA induction is a strain-specific bacterial trait.	[70]
Commensal antigens administration may help to widen the repertoire of specific Tregs for restoration and maintenance of intestinal homeostasis.	[75]
The gut SIgA targeting specific bacterial antigen can control specific bacteria growth influencing microbiota composition.	[42]
ILC3 functions as a critical regulatory checkpoint in the generation of TD colonic SIgA and maintain tissue homeostasis and mutualism with commensal microbiota.	[40]
SIgA plays a critical role in controlling gut microbial community composition and maintain a diverse and stable gut microbiota.	[89]

## References

[1] Schroeder H.W.J., Cavacini L. (2010). Structure and Function of Immunoglobulins (author manuscript). J. Allergy Clin. Immunol..

[2] Woof J.M., Ken M.A. (2006). The function of immunoglobulin A in immunity. J. Pathol..

[3] Woof J.M., Kerr M.A. (2004). IgA function—Variations on a theme. Immunology.

[4] Fagarasan S., Honjo T. (2003). Intestinal IgA synthesis: Regulation of front-line body defences. Nat. Rev. Immunol..

[5] Woof J.M., Russell M.W. (2011). Structure and function relationships in IgA. Mucosal Immunol..

[6] Gibbons D.L., Spencer J. (2011). Mouse and human intestinal immunity: Same ballpark, different players; Different rules, same score. Mucosal Immunol..

[7] Sánchez-Salguero E., Mondragón-Ramírez G.K., Alcántara-Montiel J.C., Cérbulo-Vázquez A., Villegas-Domínguez X., Contreras-Vargas V.M., del Thompson-Bonilla M.R., Romero-Ramírez H., Santos-Argumedo L. (2019). Infectious episodes during pregnancy, at particular mucosal sites, increase specific IgA1 or IgA2 subtype levels in human colostrum. Matern. Health Neonatol. Perinatol..

[8] Suzuki K., Fagarasan S. (2008). How host-bacterial interactions lead to IgA synthesis in the gut. Trends Immunol..

[9] Cerutti A., Rescigno M. (2008). The Biology of Intestinal Immunoglobulin A Responses. Immunity.

[10] Mestas J., Hughes C.C.W. (2004). Of Mice and Not Men: Differences between Mouse and Human Immunology. J. Immunol..

[11] Tao L., Reese T.A. (2017). Making Mouse Models That Reflect Human Immune Responses. Trends Immunol..

[12] Beura L.K., Hamilton S.E., Bi K., Schenkel J.M., Odumade O.A., Casey K.A., Thompson E.A., Fraser K.A., Rosato P.C., Filali-Mouhim A. (2016). Normalizing the environment recapitulates adult human immune traits in laboratory mice. Nature.

[13] Reese T.A., Bi K., Kambal A., Filali-Mouhim A., Beura L.K., Bürger M.C., Pulendran B., Sekaly R.P., Jameson S.C., Masopust D. (2016). Sequential Infection with Common Pathogens Promotes Human-like Immune Gene Expression and Altered Vaccine Response. Cell Host Microbe.

[14] Payne K.J., Crooks G.M. (2007). Immune-Cell Lineage Commitment: Translation from Mice to Humans. Immunity.

[15] Iversen R., Snir O., Stensland M., Kroll J.E., Steinsbø Ø., Korponay-Szabó I.R., Lundin K.E.A., de Souza G.A., Sollid L.M. (2017). Strong Clonal Relatedness between Serum and Gut IgA despite Different Plasma Cell Origins. Cell Rep..

[16] Kutteh W.H., Prince S.J., Mestecky J. (1982). Tissue origins of human polymeric and monomeric IgA. J. Immunol..

[17] Bunker J.J., Flynn T.M., Koval J.C., Shaw D.G., Meisel M., McDonald B.D., Ishizuka I.E., Dent A.L., Wilson P.C., Jabri B. (2015). Innate and Adaptive Humoral Responses Coat Distinct Commensal Bacteria with Immunoglobulin A. Immunity.

[18] Cerutti A., Chen K., Chorny A. (2011). Immunoglobulin Responses at the Mucosal Interface. Annu. Rev. Immunol..

[19] Suzuki K., Nakajima A. (2014). New aspects of IgA synthesis in the gut. Int. Immunol..

[20] Nochi T., Denton P.W., Wahl A., Garcia J.V. (2013). Cryptopatches Are Essential for the Development of Human GALT. Cell Rep..

[21] Spencer J., Sollid L.M. (2016). The human intestinal B-cell response. Mucosal Immunol..

[22] Suzuki K., Fagarasan S. (2009). Diverse regulatory pathways for IgA synthesis in the gut. Mucosal Immunol..

[23] Tezuka H., Ohteki T. (2019). Regulation of IgA production by intestinal dendritic cells and related cells. Front. Immunol..

[24] Sutherland D.B., Fagarasan S. (2012). IgA synthesis: A form of functional immune adaptation extending beyond gut. Curr. Opin. Immunol..

[25] Fagarasan S., Muramatsu M., Suzuki K., Nagaoka H., Hiai H., Honjo T. (2002). Critical roles of activation-induced cytidine deaminase in the homeostasis of gut flora. Science.

[26] Fagarasan S., Kawamoto S., Kanagawa O., Suzuki K. (2010). Adaptive Immune Regulation in the Gut: T Cell–Dependent and T Cell–Independent IgA Synthesis. Annu. Rev. Immunol..

[27] Reboldi A., Cyster J.G. (2016). Peyer’s patches: Organizing B-cell responses at the intestinal frontier. Immunol. Rev..

[28] Qin L., Waseem T.C., Sahoo A., Bieerkehazhi S., Zhou H., Galkina E.V., Nurieva R. (2018). Insights into the molecular mechanisms of T follicular helper-mediated immunity and pathology. Front. Immunol..

[29] Bergqvist P., Gärdby E., Stensson A., Bemark M., Lycke N.Y. (2006). Gut IgA Class Switch Recombination in the Absence of CD40 Does Not Occur in the Lamina Propria and Is Independent of Germinal Centers. J. Immunol..

[30] Berkowska M.A., Driessen G.J.A., Bikos V., Grosserichter-Wagener C., Stamatopoulos K., Cerutti A., He B., Biermann K., Lange J.F., Van Der Burg M. (2011). Human memory B cells originate from three distinct germinal center-dependent and -independent maturation pathways. Blood.

[31] Kiss E.A., Vonarbourg C., Kopfmann S., Hobeika E., Finke D., Esser C., Diefenbach A. (2011). Natural aryl hydrocarbon receptor ligands control organogenesis of intestinal lymphoid follicles. Science.

[32] Tamayo E., Alvarez P., Merino R. (2018). TGFβ superfamily members as regulators of B cell development and function—implications for autoimmunity. Int. J. Mol. Sci..

[33] Castigli E., Scott S., Dedeoglu F., Bryce P., Jabara H., Bhan A.K., Mizoguchi E., Geha R.S. (2004). Impaired IgA class switching inn APRIL-deficient mice. Proc. Natl. Acad. Sci. USA.

[34] Von Bülow G.U., Van Deursen J.M., Bram R.J. (2001). Regulation of the T-independent humoral response by TACI. Immunity.

[35] Castigli E., Wilson S.A., Garibyan L., Rachid R., Bonilla F., Schneider L., Geha R.S. (2005). TACI is mutant in common variable immunodeficiency and IgA deficiency. Nat. Genet..

[36] Gutzeit C., Magri G., Cerutti A. (2014). Intestinal IgA production and its role in host-microbe interaction. Immunol. Rev..

[37] Xiong N., Hu S. (2015). Regulation of intestinal IgA responses. Cell. Mol. Life Sci..

[38] Arnold J.N., Wormald M.R., Sim R.B., Rudd P.M., Dwek R.A. (2007). The impact of glycosylation on the biological function and structure of human immunoglobulins. Annu. Rev. Immunol..

[39] Mantis N.J., Rol N., Corthésy B. (2011). Secretory IgA’s complex roles in immunity and mucosal homeostasis in the gut. Mucosal Immunol..

[40] Melo-Gonzalez F., Kammoun H., Evren E., Dutton E.E., Papadopoulou M., Bradford B.M., Tanes C., Fardus-Reid F., Swann J.R., Bittinger K. (2019). Antigen-presenting ILC3 regulate T cell-dependent IgA responses to colonic mucosal bacteria. J. Exp. Med..

[41] Bollinger R.R., Everett M.L., Palestrant D., Love S.D., Lin S.S., Parker W. (2003). Human secretory immunoglobulin A may contribute to biofilm formation in the gut. Immunology.

[42] Joglekar P., Ding H., Canales-Herrerias P., Pasrich P.J., Sonnenburg J.L., Peterson D.A. (2019). Intestinal IgA regulates expression of a fructan polysaccharide utilization locus in colonizing gut commensal Bacteroides thetaiotaomicron. MBio.

[43] Perrier C., Sprenger N., Corthésy B. (2006). Glycans on secretory component participate in innate protection against mucosal pathogens. J. Biol. Chem..

[44] Uren T.K., Wijburg O.L.C., Simmons C., Johansen F.E., Brandtzaeg P., Strugnell R.A. (2005). Vaccine-induced protection against gastrointestinal bacterial infections in the absence of secretory antibodies. Eur. J. Immunol..

[45] Forbes S.J., Bumpus T., McCarthy E.A., Corthésy B., Mantis N.J. (2011). Transient suppression of shigella flexneri type 3 secretion by a protective O-antigen-specific monoclonal igA. MBio.

[46] Pabst O., Slack E. (2020). IgA and the intestinal microbiota: The importance of being specific. Mucosal Immunol..

[47] Lycke N.Y., Bemark M. (2017). The regulation of gut mucosal IgA B-cell responses: Recent developments. Mucosal Immunol..

[48] Benckert J., Schmolka N., Kreschel C., Zoller M.J., Sturm A., Wiedenmann B., Wardemann H. (2011). The majority of intestinal IgA^+^ and IgG^+^ plasmablasts in the human gut are antigen-specific. J. Clin. Investig..

[49] Tsui F.P., Egan W., Summers M.F., Andrew Byrd R., Schneerson R., Robbins J.B. (1988). Determination of the structure of the Escherichia coli K100 capsular polysaccharide, cross-reactive with the capsule from type b Haemophilus influenzae. Carbohydr. Res..

[50] Crivelli C., Demarta A., Peduzzi R. (2001). Intestinal secretory immunoglobulin A (sIgA) response to Aeromonas exoproteins in patients with naturally acquired Aeromonas diarrhea. FEMS Immunol. Med. Microbiol..

[51] Winsor D.K., Mathewson J.J., DuPont H.L. (1986). Western blot analysis of intestinal secretory immunoglobulin a response to Campylobacter jejuni antigens in patients with naturally acquired Campylobacter enteritis. Gastroenterology.

[52] Winsor D.K., Mathewson J.J., Dupont H.L., Winsor D.K. (1988). Comparison of serum and fecal antibody responses of patients with naturally acquired shigella sonnei infection. J. Infect. Dis..

[53] Hara S., Sasaki T., Satoh-Takayama N., Kanaya T., Kato T., Takikawa Y., Takahashi M., Tachibana N., Kim K.S., Surh C.D. (2019). Dietary Antigens Induce Germinal Center Responses in Peyer’s Patches and Antigen-Specific IgA Production. Front. Immunol..

[54] Blutt S.E., Miller A.D., Salmon S.L., Metzger D.W., Conner M.E. (2012). IgA is important for clearance and critical for protection from rotavirus infection. Mucosal Immunol..

[55] Macpherson A.J., Köller Y., McCoy K.D. (2015). The bilateral responsiveness between intestinal microbes and IgA. Trends Immunol..

[56] Bowes T., Wagner E.R., Boffey J., Nicholl D., Cochrane L., Benboubetra M., Conner J., Furukawa K., Furukawa K., Willison H.J. (2002). Tolerance to self gangliosides is the major factor restricting the antibody response to lipopolysaccharide core oligosaccharides in Campylobacter jejuni strains associated with Guillain-Barré syndrome. Infect. Immun..

[57] Turula H., Wobus C.E. (2018). The Role of the Polymeric Immunoglobulin Receptor and Secretory Immunoglobulins during Mucosal Infection and Immunity. Viruses.

[58] Li Y., Jin L., Chen T., Pirozzi C.J. (2020). The Effects of Secretory IgA in the Mucosal Immune System. Biomed. Res. Int..

[59] Vernocchi P., Del Chierico F., Putignani L. (2020). Gut microbiota metabolism and interaction with food components. Int. J. Mol. Sci..

[60] Meffre E., Wardemann H. (2008). B-cell tolerance checkpoints in health and autoimmunity. Curr. Opin. Immunol..

[61] Wu H.Y., Weiner H.L. (2003). Oral Tolerance. Immunol. Res..

[62] Eberl G. (2016). Immunity by equilibrium. Nat. Rev. Immunol..

[63] Rescigno M., Lopatin U., Chieppa M. (2008). Interactions among dendritic cells, macrophages, and epithelial cells in the gut: Implications for immune tolerance. Curr. Opin. Immunol..

[64] Zhou L., Sonnenberg G.F. (2018). Essential immunologic orchestrators of intestinal homeostasis. Sci. Immunol..

[65] Noble A., Durant L., Hoyles L., McCartney A.L., Man R., Segal J., Costello S.P., Hendy P., Reddi D., Bouri S. (2020). Deficient Resident Memory T Cell and CD8 T Cell Response to Commensals in Inflammatory Bowel Disease. J. Crohn’s Colitis.

[66] Idzko M., Hammad H., Van Nimwegen M., Kool M., Willart M.A.M., Muskens F., Hoogsteden H.C., Luttmann W., Ferrari D., Di Virgilio F. (2007). Extracellular ATP triggers and maintains asthmatic airway inflammation by activating dendritic cells. Nat. Med..

[67] Mandapathil M., Hilldorfer B., Szczepanski M.J., Czystowska M., Szajnik M., Ren J., Lang S., Jackson E.K., Gorelik E., Whiteside T.L. (2010). Generation and accumulation of immunosuppressive adenosine by human CD4+CD25highFOXP3+ regulatory T Cells. J. Biol. Chem..

[68] Li M., Zhao W., Wang Y., Jin L., Jin G., Sun X., Wang W., Wang K., Xu X., Hao J. (2020). A wave of Foxp3+ regulatory T cell accumulation in the neonatal liver plays unique roles in maintaining self-tolerance. Cell. Mol. Immunol..

[69] Bos N.A., Kimura H., Meeuwsen C.G., De Visser H., Hazenberg M.P., Wostmann B.S., Pleasants J.R., Benner R., Marcus D.M. (1989). Serum immunoglobulin levels and naturally occurring antibodies against carbohydrate antigens in germ-free BALB/c mice fed chemically defined ultrafiltered diet. Eur. J. Immunol..

[70] Yang C., Mogno I., Contijoch E., Borgerding J., Aggarwala V., Li Z., Grasset E., Helmus D., Dubinsky M., Mehandru S. (2019). Strain-level differences in gut microbiome composition determine fecal IgA levels and are modifiable by gut microbiota manipulation. bioRxiv.

[71] Wei M., Shinkura R., Doi Y., Maruya M., Fagarasan S., Honjo T. (2011). Mice carrying a knock-in mutation of Aicda resulting in a defect in somatic hypermutation have impaired gut homeostasis and compromised mucosal defense. Nat. Immunol..

[72] Kawamoto S., Tran T.H., Maruya M., Suzuki K., Doi Y., Tsutsui Y., Kato L.M., Fagarasan S. (2012). The inhibitory receptor PD-1 regulates IgA selection and bacterial composition in the gut. Science.

[73] Fernandez M.I., Pedron T., Tournebize R., Olivo-Marin J.C., Sansonetti P.J., Phalipon A. (2003). Anti-inflammatory role for intracellular dimeric immunoglobulin A by neutralization of lipopolysaccharide in epithelial cells. Immunity.

[74] Gratz I.K., Campbell D.J. (2014). Organ-specific and memory Treg cells: Specificity, development, function, and maintenance. Front. Immunol..

[75] Kuczma M.P., Szurek E.A., Cebula A., Chassaing B., Jung Y.J., Kang S.M., Fox J.G., Stecher B., Ignatowicz L. (2020). Commensal epitopes drive differentiation of colonic Tregs. Sci. Adv..

[76] Derrien M., Belzer C., de Vos W.M. (2017). Akkermansia muciniphila and its role in regulating host functions. Microb. Pathog..

[77] Smith P.M., Howitt M.R., Panikov N., Michaud M., Gallini C.A., Bohlooly-Y M., Glickman J.N., Garrett W.S. (2013). The microbial metabolites, short-chain fatty acids, regulate colonic T reg cell homeostasis. Science.

[78] Arpaia N., Campbell C., Fan X., Dikiy S., Van Der Veeken J., Deroos P., Liu H., Cross J.R., Pfeffer K., Coffer P.J. (2013). Metabolites produced by commensal bacteria promote peripheral regulatory T-cell generation. Nature.

[79] Li M., van Esch B.C.A.M., Wagenaar G.T.M., Garssen J., Folkerts G., Henricks P.A.J. (2018). Pro- and anti-inflammatory effects of short chain fatty acids on immune and endothelial cells. Eur. J. Pharmacol..

[80] Kelly D., Campbell J.I., King T.P., Grant G., Jansson E.A., Coutts A.G.P., Pettersson S., Conway S. (2004). Commensal anaerobic gut bacteria attenuate inflammation by regulating nuclear-cytoplasmic shutting of PPAR-γ and ReIA. Nat. Immunol..

[81] Kelly D., Conway S., Aminov R. (2005). Commensal gut bacteria: Mechanisms of immune modulation. Trends Immunol..

[82] Peterson D.A., McNulty N.P., Guruge J.L., Gordon J.I. (2007). IgA Response to Symbiotic Bacteria as a Mediator of Gut Homeostasis. Cell Host Microbe.

[83] Peterson D.A., Planer J.D., Guruge J.L., Xue L., Downey-Virgin W., Goodman A.L., Seedorf H., Gordon J.I. (2015). Characterizing the interactions between a naturally primed immunoglobulin a and its conserved Bacteroides thetaiotaomicron species-specific epitope in gnotobiotic mice. J. Biol. Chem..

[84] Sutherland D.B., Suzuki K., Fagarasan S. (2016). Fostering of advanced mutualism with gut microbiota by Immunoglobulin A. Immunol. Rev..

[85] Reboldi A., Arnon T.I., Rodda L.B., Atakilit A., Sheppard D., Cyster J.G. (2016). Mucosal immunology: IgA production requires B cell interaction with subepithelial dendritic cells in Peyer’s patches. Science.

[86] Palm N.W., De Zoete M.R., Cullen T.W., Barry N.A., Stefanowski J., Hao L., Degnan P.H., Hu J., Peter I., Zhang W. (2014). Immunoglobulin A coating identifies colitogenic bacteria in inflammatory bowel disease. Cell.

[87] Viladomiu M., Kivolowitz C., Abdulhamid A., Dogan B., Victorio D., Castellanos J.G., Woo V., Teng F., Tran N.L., Sczesnak A. (2017). IgA-coated E. Coli enriched in Crohn’s disease spondyloarthritis promote TH17-dependent inflammation. Sci. Transl. Med..

[88] D’Auria G., Peris-Bondia F., Džunková M., Mira A., Collado M.C., Latorre A., Moya A. (2013). Active and secreted IgA-coated bacterial fractions from the human gut reveal an under-represented microbiota core. Sci. Rep..

[89] Catanzaro J.R., Strauss J.D., Bielecka A., Porto A.F., Lobo F.M., Urban A., Schofield W.B., Palm N.W. (2019). IgA-deficient humans exhibit gut microbiota dysbiosis despite secretion of compensatory IgM. Sci. Rep..

[90] Mirpuri J., Raetz M., Sturge C.R., Wilhelm C.L., Benson A., Savani R.C., Hooper L.V., Yarovinsky F. (2013). Proteobacteria-specific IgA regulates maturation of the intestinal microbiota. Gut Microbes.

[91] Baldassarre M.E., Di Mauro A., Mastromarino P., Fanelli M., Martinelli D., Urbano F., Capobianco D., Laforgia N. (2016). Administration of a multi-strain probiotic product to women in the perinatal period differentially affects the breast milk cytokine profile and may have beneficial effects on neonatal gastrointestinal functional symptoms. A randomized clinical trial. Nutrients.

[92] Wang J.Z., Zhang Y.H., Guo X.H., Zhang H.Y., Zhang Y. (2016). The double-edge role of B cells in mediating antitumor T-cell immunity: Pharmacological strategies for cancer immunotherapy. Int. Immunopharmacol..

[93] Liu M., Sun Q., Wang J., Wei F., Yang L., Ren X. (2019). A new perspective: Exploring future therapeutic strategies for cancer by understanding the dual role of B lymphocytes in tumor immunity. Int. J. Cancer.

